# A Blockchain-IoT Platform for the Smart Pallet Pooling Management

**DOI:** 10.3390/s21186310

**Published:** 2021-09-21

**Authors:** Chun-Ho Wu, Yung-Po Tsang, Carman Ka-Man Lee, Wai-Ki Ching

**Affiliations:** 1 Department of Supply Chain and Information Management, The Hang Seng University of Hong Kong, Shatin, Hong Kong; 2 Department of Industrial and Systems Engineering, The Hong Kong Polytechnic University, Kowloon, Hong Kong; yungpo.tsang@polyu.edu.hk (Y.-P.T.); ckm.lee@polyu.edu.hk (C.K.-M.L.); 3 Department of Mathematics, The University of Hong Kong, Pokfulam Road, Hong Kong; wching@hku.hk

**Keywords:** decentralisation, pallet pooling, blockchain, Internet of things, technological eco-system, human–computer interaction

## Abstract

Pallet management as a backbone of logistics and supply chain activities is essential to supply chain parties, while a number of regulations, standards and operational constraints are considered in daily operations. In recent years, pallet pooling has been unconventionally advocated to manage pallets in a closed-loop system to enhance the sustainability and operational effectiveness, but pitfalls in terms of service reliability, quality compliance and pallet limitation when using a single service provider may occur. Therefore, this study incorporates a decentralisation mechanism into the pallet management to formulate a technological eco-system for pallet pooling, namely Pallet as a Service (PalletaaS), raised by the foundation of consortium blockchain and Internet of things (IoT). Consortium blockchain is regarded as the blockchain 3.0 to facilitate more industrial applications, except cryptocurrency, and the synergy of integrating a consortium blockchain and IoT is thus investigated. The corresponding layered architecture is proposed to structure the system deployment in the industry, in which the location-inventory-routing problem for pallet pooling is formulated. To demonstrate the values of this study, a case analysis to illustrate the human–computer interaction and pallet pooling operations is conducted. Overall, this study standardises the decentralised pallet management in the closed-loop mechanism, resulting in a constructive impact to sustainable development in the logistics industry.

## 1. Introduction

**Figure 1:**
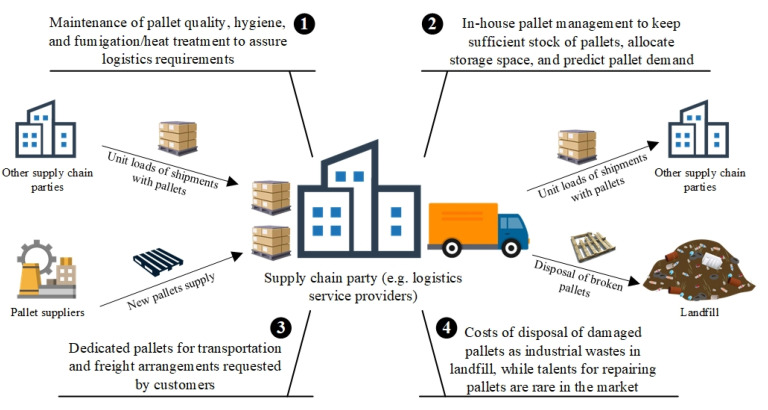
Pitfalls of traditional pallet management in the logistics and supply chain management.

In view of the research on logistics and supply chain management (LSCM), pallet management, which coordinates a sufficient number of pallets for storage and transportation, is a crucial, but under-researched area. Pallets are seen as the primary interface of a unit load, and the first line of defence to protect products (e.g., stress absorption, delivery safeguard, and ease of being handled by material-handling equipment) before reaching the designated destination [1]. Typically, material types of pallets can be softwood, hardwood, paper, metal or plastic to support diverse requirements throughout supply chains depending on cost-effectiveness, pallet lifespan, durability and damage resistance. There is no doubt that effective pallet management creates great business value in the LSCM. In view of current practice, most of the supply chain parties, such as logistics service providers, manage their own pallets in a linear flow, from sourcing, keeping pallet inventory, and using pallets in various scenarios to disposing of damaged pallets. As shown in Figure 1, four pitfalls are summarised in the current linear pallet management strategy, which is synthesised by the work [2,3,4]. Firstly, fumigation and heat treatment are two typical methods for insect control in wooden pallets under the international standards for phytosanitary measure no. 15 (ISPM-15) developed by international plant protection convention (IPPC) [2]. If the pallets are treated by fumigation, the validity period only lasts for 21 days. Therefore, supply chain parties are required to manage pallets and assure their quality cautiously for logistics operations. Secondly, in-house pallet management by supply chain parties themselves is challenging to keep sufficient stock for daily operations due to the fluctuation in pallet demand [3]. Thirdly, as the quality of pallets in the logistics industry is varied, some organisations may only accept the shipments loaded on dedicated pallets in terms of material type and quality, due to the operational constraints, prevention of insect infestation, and cargo protection. Lastly, in-house pallet management may bear additional costs on repairing and maintenance, while landfill disposal cost may incur for handling damaged and unrepairable pallets [4].

The above linear pallet management strategy may result in negative impact to sustainability on environment and business development without effective measures to handle industrial wastes of pallets, while business image and reputation can, therefore, be affected. Consequently, some researchers have explored the concepts of pallet pooling, which refers to pallet renting business to enhance the sustainability of using pallets in the LSCM [5,6,7]. Pallet management becomes a service provided by a dedicated company to cater for the requirements from routine operations and pallet maintenance. Subsequently, a closed-loop pallet management scheme inspired from the circular supply chains was proposed in order to eliminate industrial wastes by following the initiatives of “reuse”, “reduce”, and “recycle”. By doing so, a centralised service provider should play an important role in the supply chain network to supply, repair and dispose the pallets to all supply chain parties, which need to pay for the service subscription. It is deemed effective to manage pallets’ use, including pallet volume, quality, size and material types, across multiple facilities and work locations. Resources on in-house pallet management can be released to other value-added activities for the supply chain parties. Generally speaking, a concept of total pallet management is initiated to manage pallet life in the industry comprehensively. In light of the above foundation of pallet pooling in this industry, pallets as an essential asset are exchanged between supply chain parties in the closed-loop network. Although the benefits of deploying the pallet pooling strategy, particularly for business and economic sustainability in the logistics industry, are commonly understood, its implementation is not widespread. Compared with traditional pallet management practices (e.g., direct purchase from pallet suppliers), the information flow is more complicated to trace, track, and authenticate the pallets circulated in the logistics network. The lack of a standardised, reliable and secure information platform with high ease of use to operators may hinder the development of pallet pooling in the logistics industry. Furthermore, the service providers’ perspectives concern the cost-effectiveness of pallet pooling activities, referring to pallet pickup and delivery at various customer locations. When minimising operational costs, the sufficient pallet supply to end-users should be maintained to ensure the smoothness of their logistics operations. Consequently, the benefits of pallet pooling are not fully revealed in the industry, while the standardisation of pallet operations and information is still under-researched. In short, the problem statements of the existing pallet pooling strategy are as follows:

- A lack of the standardised, reliable and secure information platform for achieving pallet pooling with high ease of use;
- Difficulty in striking a balance between operational cost-effectiveness and service level in the pallet pooling scenario.

To address the above concerns, a blockchain-Internet of things (BIoT) platform to drive a smart pallet management environment is proposed, while an intelligent eco-system for the pallet management between various stakeholders, for example, pallet users, suppliers and waste handling companies, can be effectively organised. In the platform, the stock level of pallets can be automatically evaluated by the proposed platform based on the historical demand patterns and pallet management costs according to the (*R*, *Q*) replenishment policy. Subsequently, a location-inventory-routing problem for forward and reverse logistics is modelled for the above pallet pooling operations to distribute usable pallets and collect damaged and obsolete pallets back to the depots. With the aid of the proposed platform, the interaction between end-users and computers can become intelligent and automated to sustain the business operations of pallet pooling. Therefore, positive influence from the pallet pooling strategy can be leveraged to foster a sustainable and resilient logistics network.

The rest of this study is organised as follows. Section 2 synthesises state-of-the-art insights gained in pallet management modes, blockchain, IoT and logistics applications of the BIoT technology. Section 3 discloses methodological principles of constructing the pallet as a service. In Section 4, the conceptualisation of a BIoT framework and its illustration on GD-HK-MO GBA are presented. Section 5 contains the proposed framework’s academic and managerial implications, while the conclusion, limitations and future directions are summarised in Section 6.

## 2. Literature Review

In this section, the various strategies of pallet management in logistics are discussed and compared. In order to build the BIoT framework, the integration of blockchain and IoT is revealed, while its roles in the LSCM are investigated.

### 2.1. Strategies of Pallet Management

In view of the pallet management in the logistics industry, the strategies can be classified as open-loop and closed-loop pallet management systems, where the reverse pallet logistics are considered in the closed-loop system [5]. Regarding the open-loop system, the exchange of pallets among supply chain parties is performed in two modes, namely (i) single-use expendable mode and (ii) buy-and-sell mode [6]. The former represents those pallets purchased by suppliers or obtained from other companies are expendable and likely to be disposed after shipments, in which inexpensive types of pallets, such as whitewood, are utilised to result in a low purchase price of pallets. Additionally, the cost of reverse logistics can be saved in this mode, and companies can freely purchase specific sizes, quality and material types of pallets. In addition, the latter refers to pallets that are continuously sold to the next supply chain party throughout the whole supply chain until the pallets are damaged or excessive. Those obsolete pallets are re-purchased by pallet suppliers for refurbishment, reselling and disposal. Compared with the single-use expendable mode, the buy-and-sell mode is relatively environmentally friendly and sustainable to the logistics industry, where industrial wastes from pallets can be eliminated. For the closed-loop system, the concept of pallet pooling is thus introduced, where pallet pooling providers play an essential role to manage pallets, including leasing, sourcing appropriate pallet types and sizes, refurbishment, disposal, and quality control, to dedicated supply chain parties who join the network of pallet pooling [7]. It is found that the pallet pooling scheme is significant to improve logistics efficiency and operational cost-effectiveness [8]. Overall, a comparison between the above pallet management strategies presented in work [5,6,7] is presented in Table 1.

Apart from the consideration of reverse logistics and pallet ownership, the adoption of the single-use expendable mode requires huge resources to plan, source, control and manage the pallets. Although its flexibility is relatively high as there is no constraint from other supply chain parties or service providers, the environment’s sustainability and business competitiveness are the worst. The pallet pooling scheme in the closed-loop system is advantageous on resource management and sustainability. Still, the flexibility on size, quality and material types is subject to the service providers’ provision. Although several benefits are obtained from the pallet pooling scheme, two existing challenges are observed, namely (i) costly to maintain all size, material types, and quality of pallets by a pallet pooling provider, and (ii) challenging to optimise the use of leased pallets in the network due to uncertainties in the recycling process, quantity of pallets in use and pallet demand. Some scholars considered the potential of emerging information and communication technologies, such as radio frequency identification and cloud computing [9,10]. For building an effective pallet pooling strategy in the industry, a pallet pooling platform to include multiple service providers is needed to innovate the use of pallets from a physical unit load material to the Pallet as a Service (PalletaaS) by means of blockchain and IoT technologies.

### 2.2. Blockchain and the Internet of Things

Blockchain and IoT are regarded as two contemporary state-of-the-art technologies, while their hybridisation draws significant attention to create reliable, secure and trustworthy information systems [11,12]. On the one hand, IoT is an amalgamation of smart devices, machines and systems to interconnect physical objects, namely things and digital platforms over the Internet [13,14]. However, vulnerability is always one of the critical challenges in cybersecurity, and most IoT solutions are eager to embed secure and reliable mechanisms to avoid cloud attacks, for example, data tampering [15,16]. On the other hand, blockchain is a distributed database to chain the data in blocks over the peer-to-peer (P2P) network. Nevertheless, blockchain itself is ineffective for the design and development of data-intensive applications, which require huge computational power to mine and validate the blockchain [17,18,19]. Thus, the integration of blockchain and IoT, namely blockchain-IoT (BIoT), has become one of the current active research areas to overcome the challenges on storage capacity, scalability, security and data privacy in information systems [20]. Considering the BIoT, the interactions between blockchain and IoT can be classified into three types, namely (i) all IoT nodes have interacted with blockchain independently, (ii) some IoT nodes have interacted with blockchain and other IoT nodes, and (iii) some IoT nodes have interacted with blockchain, other IoT nodes, and cloud/fog/edge nodes. When the IoT nodes interact with blockchain independently, all data are stored in the blockchain as an immutable record. Still, the bandwidth and blockchain size is greatly increased for the data-intensive applications [21]. To obtain a solution with low latency, the second type of BIoT interaction is developed. Only a number of the IoT nodes are interconnected to the blockchain that communicate with other nodes in the network. Only the meaningful data are stored in the blockchain for the creation of immutable records. Thirdly, apart from interconnecting with blockchain partially, cloud/fog/edge nodes are included to establish an orchestration of real-time BIoT interaction. Such a hybrid approach fully leverages the benefits of blockchain and real-time IoT interaction. The use of cloud computing can effectively manage long-term data storage rather than storing all data in the blockchain [22]. Additionally, fog/edge computing provides additional computational power for mining and validating the blockchain, whereas real-time data can be pre-processed before loading to the blockchain [23,24]. Consequently, BIoT technology adoption can be deemed a promising approach to establish industrial applications unconventionally.

### 2.3. BIoT Technology in the Logistics and Supply Chain Management

Due to the outstanding features of the BIoT technology, it has now been applied to formulate various applications in the area of LSCM. The integration of IoT and blockchain contributes to freight tracking, environmental monitoring, carrier authentication, vehicle authentication, fast delivery and proof of delivery [25,26]. To develop BIoT applications in smart transportation and logistics, a layered architecture consisting of physical, data, network, and application layers were proposed. Furthermore, the deployment of blockchain technology in the supply chain, logistics and transport management can strengthen the capabilities of technology adoption, trust establishment between stakeholders, electronic trade facilitation, and enhanced traceability in the supply chain network [27,28]. Apart from merely technological advancements on IoT when integrating blockchain, the trust among supply chain parties is formulated as a novel value to revamp existing business models, which becomes a turning point to logistics and supply chain management. The work [29] emphasised that the logistics and supply chain collaboration was facilitated to eliminate overlapping, redundancy, and separation of logistics services in the industries. In this study, the role of the BIoT technology is further revealed in pallet management to transform the existing pallet management practice into a new, collaborative, and closed-loop pallet management strategy. Therefore, a mutually-trusted atmosphere can be established to sustain the use of pallets in the logistics industry and drive the innovation in the industries [30].

In light of on-demand services and orchestration of cyber networks, the concept of ‘anything as a service’ (ANYaaS) was developed to leverage the benefits of cloud computing in any specific domains [31]. Similarly, the ‘everything as a service’ (XaaS) was proposed to bridge the physical networks and virtualised wireless network via network virtualisation, and, thus, the provision of information and communication technology (ICT) services was the essential outcome [32,33]. In the LSCM domain, the above concepts have been widely applied to develop service-oriented business models. The work [34] proposed the ‘delivery as a service’ as a new business model to create a customer-oriented delivery process through electric and autonomous delivery vehicles. The work [35] transformed the IoT-based repelling and notifying system using blockchain to the ‘safe farming as a service’ for better cost-effectiveness and power-efficiency in agricultural supply chain management. In view of the above studies, the cyber-physical interconnection is emphasised to develop on-demand and customer-oriented services, which drives the development of a physical internet to formulate open, global and interconnected networks in the LSCM.

Furthermore, the emerging BIoT-related technologies were deployed for revamping the pallet management in the field of LSCM. To explicitly state the focus of this study, a comparative analysis with other existing pallet management solutions is conducted, as shown in Table 2. In work [9], the cloud-based pallet pooling information platform was proposed to manage the complicated information flows in the pallet pooling network. The advantages of cloud computing are leveraged to connect the intelligent services and user clients. However, the proposed platform lacks the details of pallet identification, tracking and operational modelling. Some existing work, like [10], examined the pallet pooling management from operations research. The optimisation problem was formulated to minimise the costs of operations, distribution, reposition, recycling, purchasing, loss and maintenance. Additionally, the work [36] presents the deployment of IoT technologies for tracking and monitoring the pallets and containers circulated in the logistics network. Pallets can be effectively identified with robust data storage and real-time event processing in the typical pallet management process. Nevertheless, a comprehensive pallet pooling management platform is limited in the recent literature to consider HCI, pallet identification, tracking, and real-time event handling. In contrast, the corresponding operational model on pallet pooling based on the platform is rarely discussed. Therefore, this study is motivated to design a smart pallet pooling management platform based on BIoT technologies to enhance the practicality of the pallet pooling strategy in the real-life logistics network.

## 3. Research Methodology

In this section, the research methodology of the design and development of the smart pallet pooling management system by means of BIoT technologies is outlined. The overview of the entire research methodology is provided firstly to support the BIoT platform’s design for achieving smart pallet management in the logistics network.

### 3.1. Overview of the Research Methodology

The research methodology to be presented in this study is mainly related to the design of the BIoT platform for the smart pallet pooling management, which includes the system framework design, the business logic of the proposed PalletaaS, and the optimisation model for pallet pooling. To achieve HCI in the proposed system, the platform design considers the interaction between the system and corresponding stakeholders to smoothen the operations of pallet pooling. The business logic of pallet pooling is essential to understand the interactions and business relationships of various stakeholders, such as pallet users, pallet pooling service providers, pallet manufacturers, and waste management companies. Under the systematic framework, the formulation of the optimisation problem for pallet pooling does make sense in real-life operations, resulting in enhanced practicality of the pallet pooling strategy. Subsequently, the implementation of the smart platform in the industry can be effectively facilitated.

### 3.2. Design of a BIoT Platform for Achieving Smart Pallet Management

This sub-section presents a BIoT framework as a foundation to derive pallet as a service (PalletaaS) for the LSCM based on the literature of BIoT technologies and pallet management strategies. To be specific, the system framework design, the mechanism of the PalletaaS and the operations research model are illustrated to understand the proposed platform’s functions.

#### 3.2.1. BIoT System Framework Design

**Figure 2:**
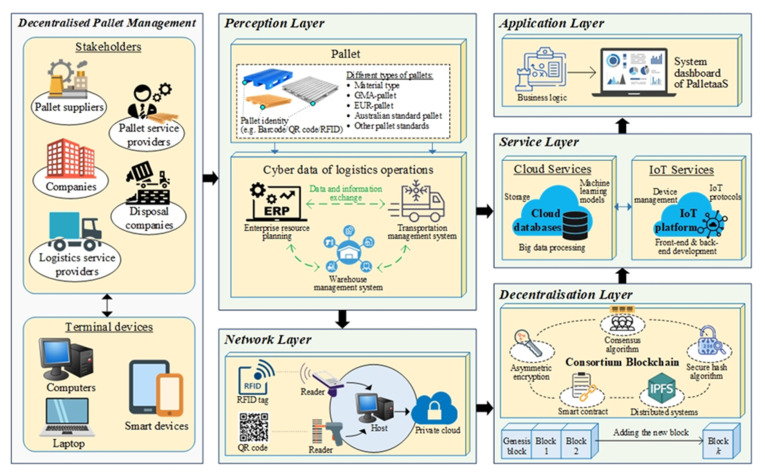
Layered architecture of PalletaaS for the decentralised pallet management.

By using blockchain and IoT for the PalletaaS, a layered architecture consisting of perception layer, network layer, decentralisation layer, service layer, and application layer is proposed, as shown in Figure 2. In the decentralised pallet management, five major types of stakeholders are considered to formulate the whole eco-system. They are pallet suppliers, pallet service providers, companies, logistics service providers and disposal companies, where companies and logistics service providers are the core pallet users in the network. Through assessing the system dashboard via smart devices, laptops and computers, the users can conveniently involve in the pallet pooling services. In order to manage the data flow of pallets in the network, a layered system framework consisting of perception layer, network layer, decentralisation layer, service layer and application layer is proposed. In the perception layer, pallets which have unique identities from barcode, quick response (QR) code, or radio frequency identification (RFID), are associated with the cyber networks together with other enterprise information systems, namely enterprise resource planning (ERP), warehouse management system (WMS), and transportation management system (TMS). Each pallet is unique, by which it contains specific status, for example material type and quality compliance, in the business network. Through the network layer, the pallet-related data, such as ownership transfer, holding duration, and pallet status, are effectively stored in the private cloud and blockchain stated in the decentralisation layer. As the blockchain is not designed for large data storage, pallet transactions between supply chain parties are mainly recorded in the blockchain to facilitate pallet tracking and traceability, while a secure pallet ownership transfer is thus built. Five essential components, including consensus algorithms, asymmetric encryption, secure hash algorithm, smart contract and distributed systems, are orchestrated to establish the consortium blockchain in the pallet pooling network. Other supplementary information for the pallet management is managed in the typical cloud services. By applying cloud and IoT services, such as data storage, data analytics, and IoT protocols managed in the service layer, the PalletaaS is built to manage the logistics network’s decentralised pallet pooling services. Based on the BIoT environment, a new business model, namely decentralised pallet pooling service, is exploited to innovate the existing pallet management practices in the industry. Like the vendor-managed inventory, the pallet pooling service providers play an essential role in managing the stock level and quality of pallets on behalf of the users who can release the resources on managing pallets in their routine operations.

In light of the merits of BIoT technology, a mutually-trusted business relationship in the PalletaaS can be formulated by utilising the consensus algorithms in the consortium blockchain, called blockchain 3.0, for developing industrial applications. The perspectives on advantages, disadvantages and representative development platforms are discussed to assist the business decision-making process, while five consensus algorithms in consortium and private blockchains are included: (i) Raft, (ii) proof of elapsed time (PoET), (iii) Istanbul Byzantine fault tolerant (IBFT), (iv) practical Byzantine fault-tolerant (pBFT), and (v) Sumeragi, as shown in Table 3 [37,38]. The above algorithms provide resource-efficient mechanisms to achieve consensus in the network, but differences on validator selection, required minimum network size, level of security, and finality efficiency are observed, which may influence the selection decision of the development platform. As the above algorithms are feasible to create consensus for the PalletaaS, the selection decision is subject to the capability of hardware and cyber security, expected network size, planned development cost and possibility of system extension. Among all three types of consensus algorithm, only the Byzantine fault tolerance-based methods consider the malicious nodes in the consensus establishment so that a strict requirement on the minimum network size is posed. In this study, the IBFT, which is adopted in the Microsoft Azure Blockchain Service, is preferred and selected to illustrate the formulation of PalletaaS. It is efficient and secure to reach the consensus in the pallet pooling network for managing the pallet transactions.

#### 3.2.2. Development of the Pallet as a Service

**Figure 3:**
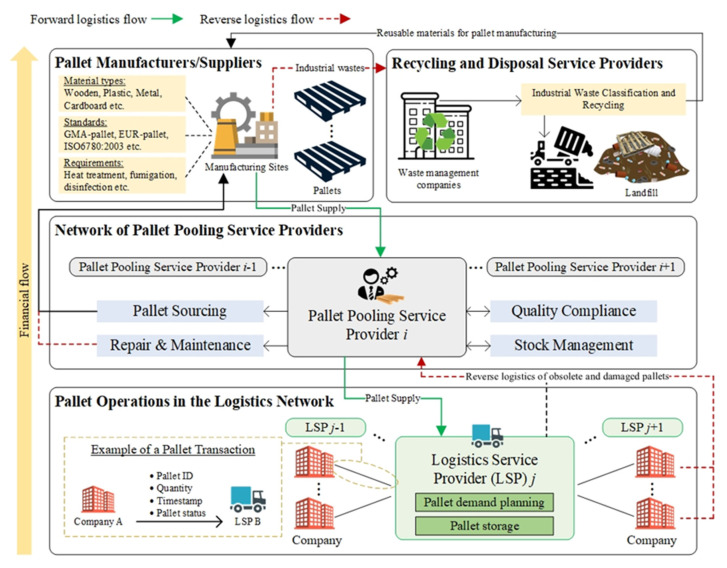
Schematic diagram of the business model of PalletaaS.

Regarding the deployment of the PalletaaS, the illustration of its business model is presented as shown in Figure 3, considering the roles of pallet suppliers, waste management companies, pallet pooling service providers (PPSPs), logistics services providers (LSPs), and other companies involving logistics activities. Several pallet manufacturers and suppliers play a vital role to produce and supply material-type-specific and standard-specific pallets to their partnered pallet pooling service providers, while pallets to be supplied should meet import and export regulations through undergoing heat treatment, fumigation and disinfection. Subsequently, the alliance of PPSPs is formed to build a P2P logistics network for pooling and managing their owned pallets. Thus, such a decentralised pallet pool contains diversified and sufficient stock of pallets for logistics operations. Typically, the PPSPs take up the role of sourcing pallets from partnered upstream suppliers, quality compliance on regulations and customer expectations, stock management to fulfil market demand, and regular repair and maintenance arrangements. Additionally, the circulation of pallets to initialise the pallet status. To participate in the above decentralised pallet pooling, companies and LSPs pay a service subscription to request on-demand services catering to their operational needs and requirements.

Consequently, the pallets offered by the PPSPs are circulated in the logistics network as the unit load material to protect cargoes. The ownership transfer of pallets in the logistics network is conducted as a pallet transaction recorded in the blockchain. The transactions concern about the sender, receiver, pallet ID, quantity, timestamp and status, which is an electronic proof of pallet transfer securely and reliably. The companies and LSPs make their own demand forecast planning on required pallet volume to handle the minimal pallets, which implies allocating minimal storage space to meet operational demand. PPSPs are responsible for coordinating regular reverse logistics to the nodes in the logistics network for the obsolete and damaged pallets while those pallets undergo the classification process. When the pallets conditions meet the expected quality standard, they are then stored in PPSPs for supporting future on-demand services. Otherwise, the damaged pallets are sent back to manufacturers and suppliers for repair and maintenance to eliminate the waste generated from the pallet usage. Any industrial wastes generated from the proposed system are sent to waste management companies to consider the recycling possibility before disposing them in the landfill.

#### 3.2.3. Location-Inventory-Routing Problem for Pallet Pooling

**Figure 4:**
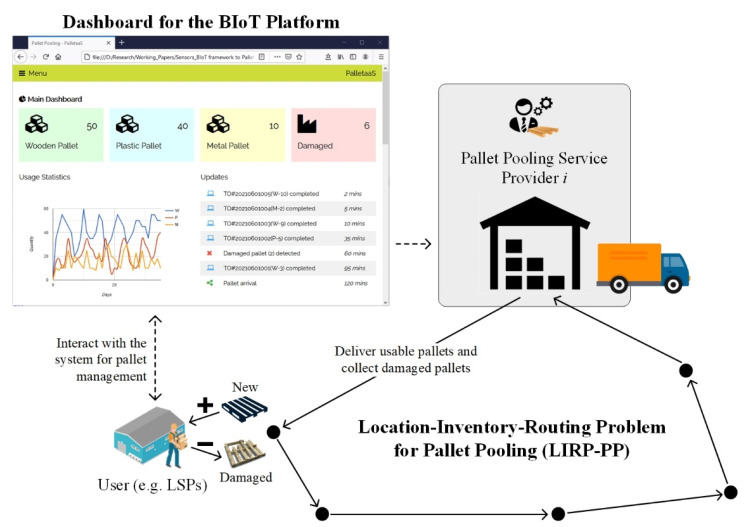
Modelling of the location-inventory-routing problem for pallet pooling.

Given a set of depots for pallet pooling service providers, the location-inventory-routing problem for pallet pooling (LIRP-PP) is formulated to optimally determine pallet quantity to users, and the paths of trucks in delivering and collect pallets [39]. As illustrated in Figure 4, the users, such as LSPs, can interact with the proposed system that connects to existing enterprise information systems to consolidate the information about the pallet usage and stock levels. After aggregating all users’ demand patterns, the pallet pooling service providers are responsible for arranging transportation services to deliver usable pallets and collecting damaged and obsolete pallets for further repair and maintenance. It is assumed that transportation routes start from and finish at the same depot, where usable pallets are delivered to users for bringing their pallet levels to the order-up-to level and damaged. Obsolete pallets are returned to the depot. Users and service providers have sufficient storage space for managing pallets at their sites. The objective of the LIRP-PP is to minimise the total inventory management cost and transportation cost of the pallet pooling service providers.

In the LIRP-PP, users adopt (*R*, *Q*) replenishment policy to manage the stock level of pallets such that the set of users I={1, 2, …, i} and depots J={1, 2, …, j} are considered. The transportation process involves a set of trucks K={1, 2, …, k} to deliver various types of pallets M={1, 2, …, m}. The pallets stored in the users’ locations are used for their routine logistics operations, where the demand pattern $d_{im}$ for specific users and pallets are assumed to be normally distributed such that $d_{im}\sim N\left(\mu_{im},\sigma_{im}\right)$. According to the (*R*, *Q*) replenishment policy [40], the (*R_im_*, *Q_im_*) policy is considered in this problem to determine the reorder point and quantity as in Equations (1) and (2), where the lead time L is uncertain and normally distributed as $L\sim N\left(\mu_{L},\sigma_{L}\right)$. The order quantity calculation follows the economic order quantity (EOQ) to minimise the annual holding and ordering cost. Once the pallet levels drop below the corresponding reorder points Rim at users’ sites, the specific order quantities *Q_im_* are placed to replenish the pallets to maintain operational effectiveness.

(1) $$R_{im}=\mu_{im}\mu_{L}+z_{\alpha }\sqrt{\mu_{L}\sigma_{im}^{2}+\mu_{im}^{2}\sigma_{L}^{2}}$$

(2) $$Q_{im}=\sqrt{\frac{2\mu_{im}c_{o}}{c_{h}}}$$

On the other hand, the pallet pooling service providers adopt the (*S −* 1, *S*) base stock policy to maintain the inventory position at the base stock level [41]. The replenishment orders from the service providers to the manufacturers are placed when the demand occurs. For the cost analysis, when using the base stock policy, the total inventory management cost (IMC) for the pallet pooling service providers is presented as in Equation (3), where the holding cost per unit h and backorder cost per unit b is defined in advance. The functions *I*(*r*) and *B*(*r*) denote the average on-hand inventory and backorders. Additionally, the optimal reorder point, namely the base stock level, has been proven such that the deduction of the optimal base stock level satisfies Equation (4), where the function G(x) represents the cumulative distribution function of demand during lead time. The value r*+1 refers to the optimal reorder point. In other words, the service providers follow the optimal inventory replenishment strategy derived by the base stock policy to maintain a specific service level to the users.

(3) IMC=hI(r)+bB(r)

(4) $$G(r^{*}+1)=\frac{b}{b+h}$$

In addition, the transportation cost (TC) between depots and users can be modelled as in Equation (5), which consists of two major components, namely (i) setup cost $c_{j}^{s}$ of using a truck at the depot, and (ii) transportation cost $c_{ij}^{t}$ of a truck on arc (i, j), where i, j∈I∪J=S and i≠j. The above considerations are then used to formulate the optimisation problem to support the daily transportation activities of the service providers.

(5) $$\mathrm{TC}=\underset{k\in K}{\sum }\underset{j\in S}{\sum }\underset{i\in J}{\sum }c_{j}^{s}x_{ijk}+\underset{k\in K}{\sum }\underset{j\in S}{\sum }\underset{i\in I}{\sum }c_{ij}^{t}x_{ijk}$$

In the mathematical modelling, the LIRP-PP is formulated to obtain the optimal transportation paths for the trucks between depots and users as follows:

(6) min {TC}

s.t.

(7) $$\underset{k\in K}{\sum }\underset{i\in S}{\sum }x_{ijk}=1,\;\forall j\in I$$

(8) $$\underset{j\in S}{\sum }x_{ijk}-\underset{j\in S}{\sum }x_{jik}=0,\;\forall i\in S\;\mathrm{and}\;\forall k\in K$$

(9) $$\underset{j\in J}{\sum }\underset{i\in I}{\sum }x_{ijk}\leq 1,\;\forall k\in K$$

(10) $$\underset{m\in M}{\sum }\underset{j\in J}{\sum }\underset{i\in I}{\sum }Q_{im}x_{ijk}\leq Q^{k},\;\forall k\in K$$

(11) $$\underset{j\in J}{\sum }\underset{i\in I}{\sum }DP_{i}x_{ijk}\leq Q^{k},\;\forall k\in K$$

(12) $$\underset{j\in U}{\sum }\underset{i\in U}{\sum }x_{ijk}\leq \left|U\right|-1,\;U\subseteq I\;\mathrm{and}\forall k\in K$$

(13) $$x_{ijk}\in \left\{0,\;1\right\}$$

Equation (5) states the objective function to minimise the transportation cost in the delivery network, while the optimal inventory replenishment policies manage the pallet levels at service providers and users. Constraint (7) denotes that each user can only be served by one truck. Constraint (8) is the equilibrium state of vehicle routes on nodes. Constraint (9) describes each arc in the delivery network can be served by one truck at most. Constraints (10) and (11) ensure the truck capability is sufficient to handle the delivery of usable pallets and collection of damaged pallets. Constraint (12) refers to the subtour elimination, in which the set U is the subset of the user set I, and the minimum number of vehicles needed to serve set U is equal to 1. Lastly, Constraint (13) shows the binary integrality to the decision variables.

## 4. Case Study

In this section, a case study is conducted to examine the performance and effectiveness of the pallet pooling approach through a numerical illustration. The process flow of using the proposed system is graphically presented to describe the various roles and procedures in the BIoT platform. In addition, the numerical illustration of the LIRP-PP is provided to suggest the vehicle routes so as to maintain the appropriate pallet level.

### 4.1. Process Flow of the Human–Computer Interaction

**Figure 5:**
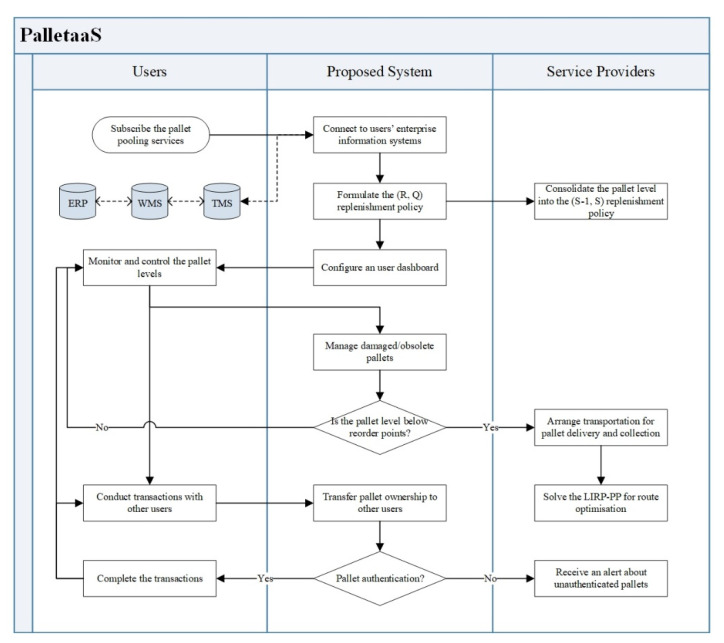
Human–computer interaction of the proposed system.

The proposed system plays an essential role between system users and service providers to facilitate the data transmission and structure the information flow. Therefore, the human–computer interaction of the PalletaaS is illustrated as shown in Figure 5. The entire service starts from the user subscription, while the interfaces between the proposed system and users’ enterprise information systems, including ERP, WMS and TMS, are built. ERP is the backbone of the logistics information systems to manage master-level information, including accounting, supplier management, inventory management, warehouse management and customer relationship management. The use of ERP solutions aims to facilitate the information flow and automate business processes between various functional departments. In order to enhance operational effectiveness and efficiency, the operations management systems, such as WMS and TMS, are incorporated to the ERP for strengthening the capability of managing day-to-day inventory movement and storage. To initialise the proposed system, the users’ customised (R, Q) replenishment policies can be formulated according to their historical data of pallet usage, which is consolidated in the (*S* − 1, *S*) replenishment policy of the service providers. In the system dashboard, users can effectively monitor and control their pallet levels, while automatic replenishment is conducted once they are below the reorder level. Subsequently, the optimisation problem of the LIRP-PP is solved to obtain the most effective path for pallet delivery and collection.

On the other hand, transactions between users can be made through the system dashboard, where the ownership transfer of the pallets can be validated when pallets are authenticated in the transaction process. If any abnormalities of the pallet authentication are recorded, corresponding alerts are sent to the service providers to spot fraudulent pallets in the market. Tag-based, location-based, and pallet-specific features are considered to build the pallet authentication mechanism to validate the pallet authenticity. Overall, the pallet management in the permissioned network can be achieved to leverage the advantages of the pallet pooling approach in the logistics industry.

### 4.2. Numerical Illustration of the LIRP-PP

Based on the customised (*R*, *Q*) replenishment policies of users and (*S* − 1, *S*) replenishment policy of the service providers, the optimisation problem of the LIRP-PP can be formulated to determine the optimal path for pallet delivery and collection. To deploy the proposed LIRP-PP, Google OR-Tools^®^ is utilised to look for the optimal route for a fleet of vehicles in the pallet pooling management. It is convenient to connect Google direction API to build the distance matrix between different users’ locations. The entire algorithm is deployed in the Python environment, where the computer configuration is as follows: Windows 10 (64-bit) with i7-6770HQ CPU@2.60GHz with 32GB installed memory.

In this numerical illustration, a depot with five available trucks to serve sixteen customers is considered. The trucks are departed at the depot to visit the users for pallet delivery and collection. Using Google Direction API and distance matrix API, the distance matrix can be formulated according to the latitude and longitude of users’ locations. On the other hand, based on the data consolidated in the PalletaaS, the pallet delivery in accordance with the (*R_im_*, *Q_im_*) replenishment policy and collection for damaged and obsolete pallets are extracted for the optimisation. As shown in Table 4, the users’ demand patterns for sixteen users C1 to C16, namely mean and variance of pallet usage, are obtained to calculate the corresponding reorder points and quantities. To be more practical, the calculated reorder points and quantities are rounded up to the next integers, namely $\left\lceil R_{im}\right\rceil$ and $\left\lceil Q_{im}\right\rceil$, where the lead time is 1, the ordering cost is HKD 50, and the holding cost/unit is HKD 2. The users with the asterisk (*) indicate that the on-hand pallet levels are below the reorder points. Thus, the corresponding reorder quantities are delivered. Together with the number of damaged and obsolete pallets at the users, total pallet delivery and pickup are summarised in Table 5. For the five available trucks, one is the 5.5 tonne truck that can load at most 1000 kg and 6 cubic metre (CBM) volume, and the rest are 9-tonne trucks to load at most 3500 kg and 18 CBM volume. Assuming that the pallet size is 120 × 80 × 12 cm^3^, the 5.5-tonne and 9-tonne trucks can load at most 52 and 156 unit of pallets, respectively. By inputting the above information in the proposed LIRP-PP, the optimal routes for the trucks can be obtained for effective delivery and pickup between the users’ locations and the depot.

In summary, there is no dropped node in this optimal delivery and pickup network such that all the demands of deliveries and pickups can be fulfilled. The five-tonne truck (T1) is not assigned to the delivery and pickup operations in the optimal solution. For the other nine-tonne trucks (T2 to T5), their routes are summarised in Table 6, where the total load and delivery quantities are presented along the optimal routes. The total distance of all routes is 45,774 m.

## 5. Discussion and Research Implications

In this section, the perspectives on the technological eco-system and BIoT advancements are qualitatively discussed, leading to sustainable development in the logistics industry. The values and research implications are then summarised to position the proposed system in the market.

### 5.1. Financial and Operational Perspectives of Adopting PalletaaS

Compared with typical pallet management strategies in the open-loop systems, pallet users must subscribe to the platform for on-demand pallet management services instead of a single pallet pooling service provider. Generally, pallet management is not the core business and a value-added activity to pallet users, but pallets are essential in logistics operations. Rather than spending resources on sourcing and managing pallets by themselves (users), the adoption of PalletaaS can leverage the advantages of blockchain and IoT technology to formulate a mutually-trusted pallet management strategy. Incorporating multiple pallet pooling service providers in the platform effectively caters to diversified users’ requirements, such as pallet type and special pallet treatments. Therefore, an internal resource for pallet management can be released to develop core businesses and value-added activities. Moreover, the operational complexity of using dedicated pallets between supply chain members can be eliminated, in which the pallet ownership transfer can be performed in the logistics network through the defined smart contracts. The documentation process of the above ownership transfer process can be digitalised and simplified. Additionally, the pallet information is traceable and transparent in the entire logistics network, which ensures that the pallet quality and standards meet the designated import and export regulations. The trust and confidence in the decentralised pallet management can be effectively cultivated to establish the process standardisation on the pallet management in the industry.

### 5.2. Sustainable Development through the PalletaaS

**Figure 6:**
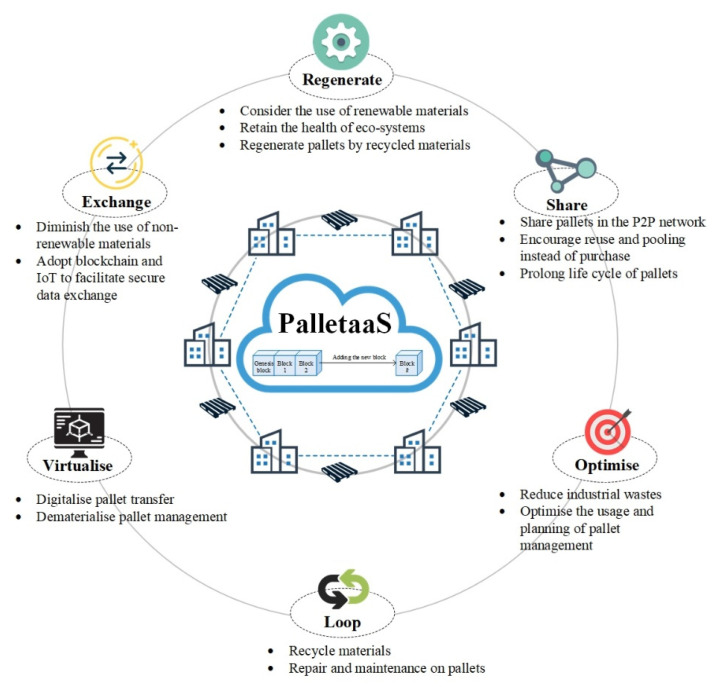
Graphical illustration of applying ReSOLVE framework in PalletaaS.

According to the ReSOLVE framework, the proposed PalletaaS can be assessed in the aspects of ‘regenerate’, ‘share’, ‘optimise’, ‘loop’, ‘virtualise’, and ‘exchange’ to align with the CE principles [42,43]. As the ReSOLVE framework is applied to boost the closed-loop supply chain development, it can be extended as the theoretical foundation of decentralised pallet management for reducing wastes, facilitating material reuse and preserving environmental-friendliness to maintain the sustainability and business longevity. The framework is applied to the PalletaaS as illustrated in Figure 6, which is customised for PalletaaS based on the ReSOLVE framework [43]. To regenerate materials and resources to the system repeatedly, the PalletaaS encourages the use of renewable materials, such as wood and cardboard, to manufacture the pallets pooled in the logistics network, which can be replenished after exploitation. Using these renewable materials for the pallet production, the health of eco-systems can be retained and sustained. As the proposed solution adopts the blockchain and IoT, the pallets are effectively shared in the P2P network to prevent excessive pallet storage and purchase in the logistics network. Additionally, the closed-loop supply chain on PalletaaS enables prolonging the pallet lifecycle with regular repair and maintenance on pallets pooled in the network. Based on the foundation built by the BIoT system, the optimisation on pallet usage can also be achieved to minimise industrial wastes and maximise the pallet management in LSPs to enhance sustainability in the whole logistics industry. When integrating forward and reverse logistics in the supply chain network, a closed-loop supply chain for pallets is formulated, where obsolete and damaged pallets are collected by PPSPs for proper repair and maintenance so as to promote reuse instead of disposal. With the aid of BIoT technology, the entire pallet management is, therefore, digitalised and virtualised, including the process of pallet transfer between various stakeholders, pooling diversified pallets in the P2P network, and managing a large group of PPSPs, LSPs and other relevant companies. The benefits of blockchain and IoT are leveraged and fully revealed in the PalletaaS to facilitate data exchange and pallet sharing in the industry. As using renewable pallets can eliminate the waste handling cost and create a positive image on the companies, incentives on using renewable pallets can be provided to reduce non-renewable materials in the market. Through the qualitative discussion assisted by the ReSOLVE framework, the PalletaaS aroused by the BIoT technology overcomes the operational challenges of using pallets in the industry and generates a positive impact on sustainability, which is regarded as a regarded as an eco-innovation concept in the logistics industry.

### 5.3. Research Implications and Values

In this section, the lessons learnt from the conceptual system of decentralised pallet management are generalised as two theoretical implications, namely integration between blockchain and IoT and process standardisation. This research represents a vision of formulating a future pallet management strategy in LSCM to drive internal and external business values.

Going from the foundation of the proposed framework, new business models through the integration of blockchain and IoT are cultivated to leverage the prestige advantages of data acquisition and cybersecurity. The theory of ubiquitous information systems (UIS) which refers to the adoption of information system thinking to create people- and process-driven solutions, can be further extended by embedding the secure and mutually-trusted mechanism for data management. In particular, to PalletaaS, the trade and transaction of pallets in the logistics network can be securely chained to show the historical track records throughout the entire lifecycle. More importantly, Byzantine nodes that might perform malicious actions to the network can be addressed by considering the Byzantine fault tolerance in the system design, which is deemed an enhancement on the typical IoT systems. In this study, the concept of consortium blockchain, which is regarded as the blockchain 3.0, is investigated [44], while the synergy of the consortium blockchain and IoT is explored to not only create a reliable systematic solution to the industry but also build the trust and process standardisation on the complicated business processes and decisions. With the above BIoT foundation, the second theoretical implication of this research is that standardisation of pallet management process is achieved through the PalletaaS. Instead of the open-loop system, the proposed PalletaaS leverages the potential of pallet pooling in a closed-loop logistics system, where a platform incorporating the concept of sharing economy is built to include verified PPSPs, LSPs and other supply chain parties. Additionally, the business process re-engineering on pallet management is achieved to re-define the usage and lifecycle management of pallets in the industry. Through such a platform-based solution to the industry, high diversification on pallets and management strategies, but in a standardised business process, can be obtained to satisfy different customers’ and market needs on pallets deemed the backbone to the logistics and supply chain activities.

From the perspective of business values, the PalletaaS built on the BIoT foundation provides the ability to track and trace pallet usage in the logistics industry and facilitates the lifecycle assessment on pallets to evaluate the environmental impact, cost-effectiveness and operational effectiveness on different kinds of pallets. Subsequently, the carbon footprints on pallet usage can be investigated to facilitate sustainable development in the logistics industry. Apart from analysing the pallet itself, the user behaviour on pallet usage can be investigated to analyse the pallet demand in the network, average pallet life span handled by various users, logistics activity network, and so on. The transactions and shipment volumes between LSPs and supply chain parties can be dug out, which implies as to the degree of bond in the business network. On the other hand, thanks to the immutable and secure mechanism from the BIoT technology, trust can be effectively established through the high level of data accuracy, privacy and confidentiality on the consortium blockchain. Risks of system vulnerability and data tampering by malicious nodes are relatively low compared with typical IoT systems. Consequently, the research of the BIoT technology enables the rooms of revamping traditional business models to a closed-loop, mutually trusted and standardised business model.

## 6. Conclusions

Integration of blockchain and IoT is a significant research trend in recent years, which cultivates many novel business models through leveraging the benefits of reliable data acquisition and robust cybersecurity on industrial applications. This study revamps the existing pallet management which is regarded as a backbone to the LSCM through the combination of consortium blockchain, namely blockchain 3.0, and IoT. Subsequently, the conceptual technology framework on PalletaaS with the layered architecture is proposed to design a closed-loop, mutually trusted and standardised systematic solution, as a novel pallet management strategy, in the logistics industry. To facilitate the pallet delivery and pickup in the PalletaaS, the LIRP-PP is proposed in this study to optimise the operations of the pallet pooling strategy. In order to ensure the alignment with CE principles, the evaluation of the proposed system was conducted by considering the ReSOLVE framework, and, thus, it is found that the PalletaaS designed in the closed-loop system provides the constructive impact on industrial waste reduction, use of renewable materials and dematerialisation on pallet management process.

Consequently, this study explores a new business synergy through the BIoT technology to facilitate the decentralisation of pallet management. To the best of our knowledge, a BIoT-driven pallet pooling management platform is rare in the recent literature and industries in the context of logistics and supply chain management. Subsequently, the values of the pallet pooling strategy are not fully revealed in the real-life logistics network, even though several benefits of pallet pooling, such as better economic and environmental sustainability, have been commonly recognised. With the aid of the smart pallet pooling management platform developed by using BIoT technologies, the pallet pooling strategy’s practicality is further enhanced to facilitate the adoption of the pallet pooling strategy for most pallet users in the logistics network.

This study includes two limitations that may motivate future research. Firstly, the technical details on developing an information system based on PalletaaS are not comprehensively discussed, for example, data structure, selection of IoT nodes, and formulation of the BIoT system. Secondly, although the industrial challenges and prestigious technologies inspire this study, the stakeholders’ perception of the proposed solution is not covered. The recent research studies assume the technology acceptance on BIoT technology. In the future, an enterprise information system for decentralised pallet management can be developed to formulate a system-wide solution for the PalletaaS. More details on the systematic process flow and data management can be presented to promote the concept of PalletaaS in the industry and verify the technical feasibility of the proposed solution. In addition, such a novel decentralised pallet management strategy can facilitate more empirical studies to investigate the evolution of pallet management strategies in the logistics industry. Challenges to implementing BIoT technology in the industries can be dug out to contribute to other BIoT-driven business applications. As blockchain technology can be seen as a mature tool in cryptocurrency but a new element to industrial applications, more research results can thus support the design and development of BIoT-centric business models in the near future.

## Figures and Tables

**Table 1:** Comparison of various pallet management strategies.

	Open-Loop System		Closed-Loop System
	Single-Use Expendable Mode	Buy-and-Sell Mode	Pallet Pooling
Reverse logistics	No	Yes, obsolete and damaged pallets are sold back to suppliers.	Yes, service providers arrange the reverse logistics regularly to collect obsolete and damaged pallets.
Pallet ownership	User	User	Service Provider
Resource management	Difficult. Companies have to manage all pallets by themselves, such as sourcing, storage and disposal, etc.	Moderate. Companies need to manage all pallets similar to single-use expendable mode, except disposal arrangement.	Easy. Pallet management is assisted by service providers, while companies merely handle on-demand pallets.
Flexibility	High. Companies can source and purchase any types and standards of pallets for their operations.	Medium. Companies need to consider the ability to sell used pallets to the next company.	Medium. Flexibility of pallets, including types and standards, is depended on the coverage of service providers.
Sustainability	Low, due to direct disposal of obsolete and damaged pallets.	Medium, due to the likelihood of excessive number of pallets in the industry.	High, due to the adoption of circular economy principles (reuse, reduce, and recycle) by service providers.

**Table 2:** Comparison with existing pallet management solutions.

	Work [9]	Work [10]	Work [36]	This Study
Technology & techniques	Cloud computing	Optimisation	IoT; cloud computing	IoT; blockchain; cloud computing; optimisation
Concerns on HCI	✓	✕	✕	✓
Pallet identification	✕	✕	✓	✓
Pallet tracking	✕	✕	✓	✓
Real-time event handling	✓	✕	✓	✓
Pallet pooling	✓	✓	✕	✓
Operational modelling	✕	✓	✕	✓

**Table 3:** Comparison of consensus algorithms for developing consortium blockchains.

	Raft	PoET	IBFT	pBFT	Sumeragi
Representative Platforms	Hyperledger Fabric, IBM Blockchain	Hyperledger Sawtooth	Azure Blockchain Service	Hyperledger Sawtooth	Hyperledger Iroha
Validator	Elected by voting	Dynamic	Dynamic	Static	Partitioned and dynamic
Foundation	Crash fault tolerance	Probabilistic selection	Byzantine fault tolerance		
Min. network size (No. of nodes)	2*N*+1	-	3*F*+1		
Advantages	High efficiency in blockchain finality, cost-effectiveness for development	High randomness in validator selection; High scalability	High efficiency in blockchain finality		
Disadvantages	Assumption of no malicious node	Delayed finality; requiring specialised hardware security	Comparatively long time to reach consensus in a large network		

Remark: *N* is the number of crashed/disappeared, but malicious nodes; *F* is the number of malicious nodes.

**Table 4:** Reorder points and quantities for sixteen users in the PalletaaS.

	Wooden Pallet				Plastic Pallet				Metal Pallet			
	Mean	Var	R	Q	Mean	Var	R	Q	Mean	Var	R	Q
C1	24	18	31	35	23	7	28	34	14	5	18	27
C2 *	16	16	23	29	30	13	36	39	10	5	14	23
C3	28	20	36	38	22	12	28	34	19	9	24	31
C4	19	16	26	31	21	8	26	33	19	10	25	31
C5 *	20	12	26	32	21	10	27	33	14	10	20	27
C6	16	16	23	29	20	11	26	32	18	6	23	30
C7	28	5	32	38	10	5	14	23	11	7	16	24
C8 *	20	17	27	32	19	14	26	31	11	10	17	24
C9 *	18	10	24	30	18	14	25	30	15	5	19	28
C10 *	28	20	36	38	15	7	20	28	15	8	20	28
C11 *	19	6	24	31	28	7	33	38	20	7	25	32
C12	17	8	22	30	28	13	34	38	14	5	18	27
C13 *	10	19	18	23	18	9	23	30	19	8	24	31
C14 *	12	6	17	25	24	12	30	35	17	8	22	30
C15 *	20	16	27	32	28	6	33	38	12	8	17	25
C16	30	18	37	39	21	15	28	33	20	5	24	32

Remark: Users marked with the asterisk (*) suggest that their on-hand pallet levels are below the reorder points.

**Table 5:** Deliveries and pickups at users’ locations.

	Wooden Pallet	Plastic Pallet	Metal Pallet	D&O
	Delivery	Delivery	Delivery	Pickup
C1	0	0	0	20
C2	29	0	23	0
C3	0	0	0	10
C4	0	0	0	5
C5	32	33	27	0
C6	0	0	0	25
C7	0	0	0	15
C8	0	31	24	0
C9	0	30	0	0
C10	38	28	28	0
C11	31	38	32	0
C12	0	0	0	35
C13	23	30	0	0
C14	25	35	0	0
C15	32	0	0	0
C16	0	0	0	5

**Table 6:** Results of solving the LIRP-PP for the pallet delivery and pickup.

	Optimal Routes	Distance (m)
T1	N/A	0
T2	Depot (113, 113) → C14(113, 113) → C13 (53, 53) → C12 (0, 0) → C6 (35, 0) → Depot (60, 0)	8226
T3	Depot (156, 156) → C9 (156, 156) → C10 (126, 126) → C15 (32, 32) → Depot (0, 0)	13,169
T4	Depot (153, 153) → C2 (153, 153) → C1 (101, 101) → C11 (121, 101) → Depot (20, 0)	11,057
T5	Depot (147, 147) → C8 (147, 147) → C4 (92, 92) → C5 (97, 92) → C7 (5, 0) → C16 (10, 0) → C3 (20, 0) → Depot (30, 0)	13,322

## Data Availability

The data presented in this study are available on request from the corresponding author. The data are not publicly available due to confidentiality of the case company.
